# Child Deaths From Injury in the Special Wards of Tokyo, Japan (2006–2010): A Descriptive Study

**DOI:** 10.2188/jea.JE20130060

**Published:** 2014-05-05

**Authors:** Hideto Suzuki, Wakako Hikiji, Takanobu Tanifuji, Nobuyuki Abe, Tatsushige Fukunaga

**Affiliations:** Tokyo Medical Examiner’s Office, Tokyo Metropolitan Government, Tokyo, Japan

**Keywords:** child fatality review, injury, mortality statistics, prevention

## Abstract

**Background:**

There has been increasing interest in the formal review of child deaths in Japan. In this study we examined the causes and scene information regarding child deaths from injury in Tokyo, the capital of Japan, as preparation for implementation of a full-scale review of child deaths.

**Methods:**

Documents on deaths from injury (excluding homicides) investigated by the Tokyo Medical Examiner’s Office during the period from 2006 through 2010 were reviewed. Deaths of children younger than 18 years (*N* = 217) were selected as the study sample. We examined the cause of and information on the death and were particularly interested in whether a case had preventable factors.

**Results:**

Overall, 67% of the cases were deaths from unintentional injury. The main cause of death among children younger than 1 year was asphyxia, and the proportions of deaths from traffic accidents were higher in older age groups. Thirty percent of deaths from injury were due to suicide, and all cases of suicide were among children older than 10 years. Although analysis of preventable factors was difficult in some cases, owing to limited information on the death scene, 87% of deaths from unintentional injury, excluding those involving traffic accidents, had preventable factors.

**Conclusions:**

Most unintentional child deaths from injury appear to be preventable. Development of a system to collect detailed information on the scene at the time of death will help decrease child deaths in Japan.

## INTRODUCTION

Although Japan has a lower infant mortality rate than many developed countries, the mortality rate for children aged 1 to 4 years is relatively high.^1^^,^^2^ It was second highest among 14 developed countries in 2001^1^ and third highest during the period 2000–2005.^2^ Several reasons for this relatively high mortality rate among children aged 1 to 4 years have been suggested, including injury,^1^ insufficient emergency medical services,^1^ and severe underlying disease in children older than 1 year. However, the actual reasons are unknown because of the difficulty in collecting data from death certificates and death scene investigations, which are necessary for the detailed analysis of child deaths in Japan.^3^

A possible solution to these challenges is to establish a review program for child deaths, in which a team systematically assesses the cause and preventability of all child deaths and investigates the epidemiology of such deaths. Such a program was initially developed in the United States over 30 years ago because of concerns regarding the underreporting of deaths from child abuse^4^ and has been successful in identifying and reducing preventable deaths.^5^^,^^6^ Recently, there have been calls to start a similar program to review child deaths in Japan, but there are many problems, such as data collection and insufficient legislation, that could delay the start of a full-scale review system.

As is the case for the review of child deaths, the Japanese system for investigating deaths remains in the developmental stage.^7^^,^^8^ Only 5 Japanese cities have a medical examiner system in place. In other areas, postmortem examination is primarily conducted by medical practitioners who are not familiar with forensic medicine, and autopsy rates are lower in these areas.^8^^–^^10^ The metropolis of Tokyo, the capital of Japan, has 23 special wards and is the largest city with a medical examiner system,^8^ which facilitates collection of information on a large number of child deaths from injury. Unintentional injury is the leading cause of death annually among 1- to 4-year-olds (*n* = 151, 16.2% of a total of 932 deaths in 2010), 5- to 9-year-olds (*n* = 125, 26% of a total of 480 deaths in 2010), and 10- to 14-year-olds (*n* = 121, 21.9% of a total of 553 deaths in 2010); suicide has been the leading cause of death among 15- to 19-year-olds (*n* = 451, 31.7% of a total of 1422 deaths in 2010).^11^ However, neither the census reports nor the published literature provide a detailed analysis, including an analysis of preventability. Therefore, we used the reports of medical examiners to investigate the manner/cause and circumstances of child deaths from injury in Tokyo. The purpose of this study was to conduct an initial review of child deaths in Japan in order to identify recent trends in child death from injury in Japan and to estimate the proportion of cases with preventable factors.

## METHODS

### Study sample

In Japan, physicians must report to the police all unnatural deaths (ie, sudden unexpected death from disease, death from injury, and death of unknown cause). When an unnatural death occurs, the police perform a death scene investigation. After that, medical examiners or physicians entrusted by the police perform a postmortem examination, with or without an autopsy. The manner or cause of death is determined by the result of this examination and a police investigation of the scene of death.

All unnatural deaths that occur in the special wards of the Tokyo metropolis are reported to the Tokyo Medical Examiner’s Office. Unnatural deaths constituted 19.3% of total death in this area during the period 2006 through 2010, and the documentation of unnatural deaths handled by the Tokyo Medical Examiner’s Office in 2006–2010 was reviewed. Deaths in individuals younger than 18 years in which the manner of death was listed as death from injury were selected as the study sample (*N* = 217). The sample was classified into 5 age groups, as in a previous study (ie, age <28 days, 28–365 days, 1–4 years, 5–9 years, 10–14 years, and 15–17 years).^6^ The documents available for review included death certificates, medical examiner reports on the postmortem examination, autopsy reports (if performed), and summaries of death scene investigations by the police. Cases of homicide or suspected homicide were excluded from this study because such cases are transferred to the forensic division of the medical departments of universities for a judicial autopsy, and it is difficult to obtain the results of autopsies in such cases.

### Classification of manner or cause of death

The manner of death from injury was classified as unintentional, intentional (suicide), or undetermined. The cause of unintentional death was classified according to the type of death certificate in Japan (eg, traffic accident, falling, drowning, fire, asphyxia, poisoning). Census data in 2006–2010^11^ were used for comparison of cause of death between the study sample and the general Japanese population.

### Analysis of preventable factors

After reviewing all the documents, 3 medical examiners (H.S., W.H., and T.F.) assessed whether there were identifiable failures (eg, lack of supervision, unsafe sleep environment) in the child’s direct care by any agent, including the parents, with direct responsibility for the child. The presence of design failures, dilapidation of barriers, or inadequate maintenance by agencies responsible for public safety was also assessed.

## RESULTS

**Figure 1.**
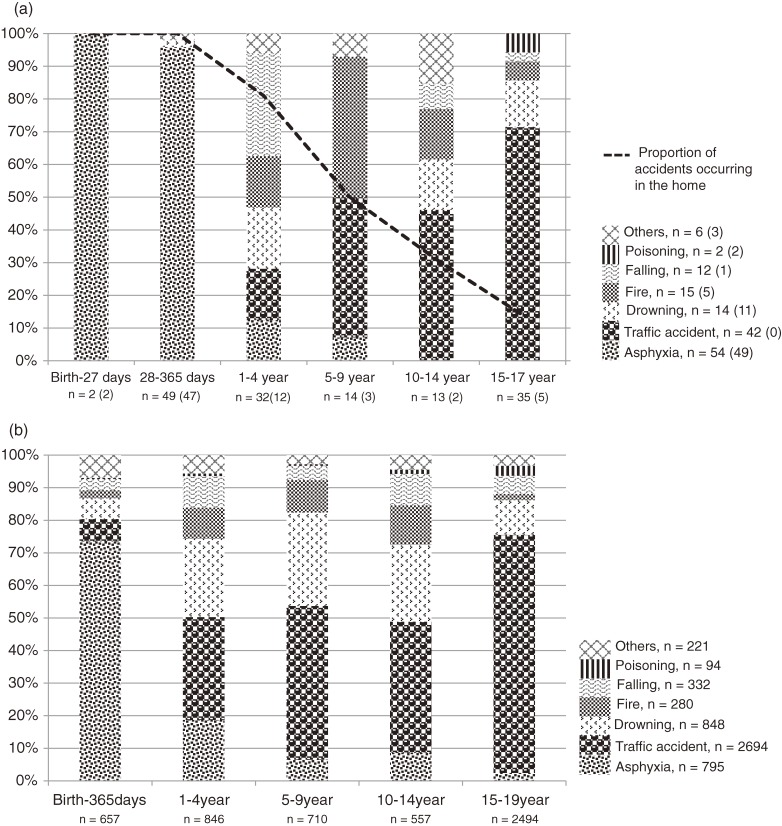
Cause of death from unintentional injury according to age. a. Data from the special wards of the Tokyo metropolis. Total number of deaths (number of autopsied cases). b. Census report data for all of Japan.

In this descriptive study, the most frequently represented age group in the sample was 15 to 17 years (*n* = 87), followed by the age group 28 to 365 days (*n* = 50) (Table 1). Death from unintentional injury constituted 67% of all cases, and causes of death from unintentional injury varied by age group (Figure 1a). Most cases of poisoning, drowning, and asphyxia were diagnosed during autopsies because it was difficult to determine these causes of death by external examination or postmortem imaging. Most deaths in infants younger than 1 year were caused by asphyxia, and deaths from traffic accidents appeared to be more common in older age groups. Similar tendencies were seen in the census report (Figure 1b). Regarding accident location, the home was a frequent place of injury in younger age groups (Figure 1a). Suicide constituted 30% of all deaths from injury, and the children were all older than 10 years (Table 1).

**Table 1.** Manner of death, according to age group

Manner of death	Age						

	Birth-27 days	28–365 days	1–4 years	5–9 years	10–14 years	15–17 years	Total
Unintentional	2 (2)	49 (47)	32 (12)	14 (3)	13 (2)	35 (5)	145 (71)
Suicide	0	0	0	0	15 (0)	51 (2)	66 (2)
Undetermined	0	1 (1)	0	0	4 (0)	1 (1)	6 (2)
Total	2 (2)	50 (48)	32 (12)	14 (3)	32 (2)	87 (8)	217 (75)

Parentheses indicate number of autopsied cases.

Asphyxia was the leading cause of death from unintentional injury, followed by traffic accidents. Among the deaths from asphyxia (*n* = 54), 50 (93%) happened in the home while the child was asleep and 48 (89%) were in infants younger than 1 year. Among the deaths while sleeping (*n* = 50), unsafe sleep conditions (eg, co-sleeping, sleeping in a facedown position) were noted in 40 cases (80%). Preventable factors (eg, unsafe sleep conditions, lack of supervision) were identified in 46 cases of death from asphyxia (85%).

The victims of traffic accidents (*n* = 42) included 15 pedestrians (36%), 12 motorcyclists (29%), 8 passengers (19%), and 7 bicyclists (16%). Preventable factors were identified in 19 cases (45%) of death from traffic injury, as shown in Table 2. However, it was difficult to identify preventable factors in the other 23 cases (55%) because of insufficient information about the accidents.

**Table 2.** Preventable factors for deaths from traffic accidents

Factor	Number
Alcohol	4
Reckless driving	4
Running a red light	3
Speeding	2
Driver inexperience	2
Lack of supervision	2
Others	2

All deaths from fire (*n* = 15) happened in the home. Playing with fire was regarded as a cause of death in 6 cases (specifically, playing with a cigarette lighter in 5 cases), and careless handling of a cigarette by parents was identified in 3 cases. Preventable factors were identified in all cases and included lack of supervision (8 cases) and careless handling of fire (11 cases).

Death from drowning (*n* = 14) was divided into 2 groups according to accident location. Eight cases happened in a bathroom of the home (6 children were younger than 5 years) and 6 cases occurred at rivers (5 children were older than 14 years). Preventable factors were identified in 12 cases (86%), as shown in Table 3.

**Table 3.** Preventable factors for death from drowning

Factor	Number
Lack of supervision	6
Access to water	5
Swimming at night	3
Alcohol	1

More than 1 factor may have been identified for a death.

Among the deaths from falling (*n* = 12), 10 children (83%) were younger than 5 years, and 11 children (92%) fell from a window or a balcony in the home. Preventable factors were identified in 11 cases (92%), as shown in Table 4.

**Table 4.** Preventable factors for death from falling

Factor	Number
Lack of supervision	9
Access to window	6
Concrete floor	1
Others	1

More than 1 factor may have been identified for a death.

Among the deaths caused by suicide (*n* = 66), 27 children (41%) killed themselves by jumping from heights, and 26 children (39%) hanged themselves. Twenty-six children (39%) had received prior mental health diagnoses, and depression was suspected in 14 children who had not consulted a mental health professional (21%). Table 5 shows several factors, identified in 39 cases (59%), that may have contributed to the children’s mental health status before suicide. Analysis of preventable factors was difficult in most cases because of lack of detailed information, such as possible motives for suicide and presence of strained relationships between family members.

**Table 5.** Factors that may have contributed to a child’s despondency before suicide

Factor	Number
Failure at school	22
Family discord	14
Recent break up with boyfriend or girlfriend	5
Recent argument with boyfriend or girlfriend	3
History of alcohol use	2
Victim of bullying	1

More than 1 factor may have been identified for a death.

## DISCUSSION

To our knowledge, this is the first review of child deaths in Japan to use medical examiner records. A previous study in the United States showed that 91% of deaths attributable to unintentional injuries were considered preventable.^4^ Because 75% of all unintentional deaths (87% of deaths due to causes other than traffic injuries) had some preventable factor in this study, most unintentional child deaths in Japan may be similarly preventable.

A characteristic distribution of the cause of death from unintentional injury was observed according to age, both in this study and the census report (Figures 1a and 1b). Asphyxia was the leading cause of death in infants, and traffic accidents were the most common cause of death by injury in children aged 1 to 14 years. These findings agree with data from the United Kingdom,^12^ Estonia,^13^ Canada,^14^ and New Zealand.^15^ The results of this study clearly show that is important to develop measures that reduce accidental deaths of children, namely, by preventing (1) infant deaths due to asphyxia while sleeping (eg, teaching parents to avoid co-sleeping, putting the infant to sleep in a face-up position), (2) accidents that occur inside the home for children under school age (eg, restricting access to water, windows, and devices such as lighters), and (3) traffic accidents involving school children.

Development of effective suicide prevention strategies is complex and difficult, but early recognition of depression and better access to mental health services are important. There was a suspicion of depression, but no contact with mental health providers, among 21% of the suicides in this study, and 60% of the suicides had factors that may have contributed to despondency before suicide.

Although a previous study found that death certificates used in child mortality data were frequently completed incorrectly,^4^^,^^16^ we found no errors in death certificates, perhaps because the certificates in this study were written by well-trained medical examiners rather than by general physicians. A review of child deaths should begin in areas where a medical examiner system is in place and correct mortality statistics can be obtained.

Analysis of preventable factors before death was difficult in some cases, especially for those involving traffic accidents and suicides, because of limited information on the death scene. In addition to correct determination of cause of death, the establishment of a system to collect detailed information regarding the death is necessary in order to assess preventability and for a full-scale review of child deaths to be most useful. Regarding transport-related deaths, it was reported that the extent of the decline in deaths among children and adolescents younger than 18 years was not as great as that among other age groups, despite the overall decline in transport-related deaths in other countries.^17^ Special attention should thus be paid to collection of traffic accident information in full-scale reviews of child deaths.

We believe that our results are an accurate summary of child deaths from injury in Japan. However, differences in cause of death were noted between the study sample and the census report, such as the proportions of falling among 1- to 4-year-olds and drowning among 5- to 9-year-olds (Figure 1a and 1b). However, this study was based on data from a large city in Japan, where death from drowning in rivers is rare.

The purpose of a program to review child deaths is to reduce child fatalities, through community-based prevention and data-driven recommendations for legislation and public policy.^4^ Local teams should review reported deaths to develop prevention strategies that best meet the needs of each community. To develop a full-scale review of child deaths throughout Japan, the death investigation system needs to be improved (eg, by implementing a medical examiner system). Further, all professionals involved in child deaths should examine and record any circumstances surrounding the death from the perspective of prevention.

This study has some limitations. First, a child fatality review team (CFRT) includes not only a medical examiner but also members of various professions, such as a local representatives from law enforcement and the county health department and a psychiatrist.^4^^,^^6^ Second, a CFRT has the authority to request and receive any documents, in addition to the medical examiner’s report, that might provide insight into the child’s death.^4^ The lack of these elements, and insufficient information on the circumstances of deaths, may have hindered identification of preventable factors. It is preferable to review traffic accidents deaths with the traffic police officers in charge of the accidents, and death from suicide should be reviewed with the help of psychiatrists and psychologists.

In conclusion, this study revealed several features of child deaths from injury in Tokyo, some of which have been identified in other countries. Most unintentional deaths from injury may be preventable, and the establishment of a system for collecting detailed information on the death of a child—eg, by improving the death investigation system throughout Japan and by better communication between clinicians and medical examiners—will contribute to the development of effective measures for reducing future child deaths in Japan.
